# Awareness of First Aid Management of Epistaxis in Children Among Parents in Arar, Saudi Arabia

**DOI:** 10.7759/cureus.49557

**Published:** 2023-11-28

**Authors:** Yahia Abdelgawad Elsayed Elboraei, Mada Muteb Alanazi, Bishri Fawzan Almesned, Wujud Khalid Alanazi, Dhai Nasser Almutairi, Iftikhar Lafi N Alanazi, Ghadah Khalid H Alanazi, Manal S. Fawzy

**Affiliations:** 1 ENT, Faculty of Medicine, Northern Border University, Arar, SAU; 2 College of Medicine, Northern Border University, Arar, SAU; 3 Biochemistry, Faculty of Medicine, Northern Border University, Arar, SAU; 4 Medical Research Unit, Faculty of Medicine, Northern Border University, Arar, SAU

**Keywords:** saudi arabia, parents, first-aid management, awareness, epistaxis

## Abstract

Background: Epistaxis is a relatively common condition, particularly among children, necessitating that parents be well informed about its effective management.

Objectives: This study aims to explore the current level of awareness among parents in the Arar region, Saudi Arabia, regarding the first aid management of epistaxis and to highlight the importance of education in empowering parents to handle such situations.

Methods: A cross-sectional study was conducted using a survey distributed among the Arar population (aged >18 years) who had at least one child and were willing to participate. Data were collected between mid-July and the end of September 2023 using a self-administered questionnaire, which included a consent form, sociodemographic and background items, and epistaxis knowledge-related questions.

Results: A total of 342 participants (27.8% males) completed the questionnaire. It was observed that 47.4% of the participants' children had experienced epistaxis. Only around half of them (n=84; 51.9%) had received first aid management for epistaxis, and only 40.4% of the parents correctly identified all the necessary steps for managing it through first aid. There was inadequate knowledge regarding the causes/risk factors and appropriate first aid techniques for epistaxis. Certain sociodemographic factors were significantly associated with better knowledge of first aid management of epistaxis, such as female gender (p = 0.003), older participants (p = 0.002), and a higher educational level (p = 0.001).

Conclusion: The study found low awareness of first aid management of epistaxis among parents residing in Arar, Saudi Arabia. Factors related to the demographic characteristics of the study participants were associated with this level of knowledge. These findings emphasize the need to improve awareness about first aid management of epistaxis, particularly among younger individuals, males, and those with limited education. Effective interventions should be developed to enhance first aid training, considering the specific risk factors associated with epistaxis.

## Introduction

Epistaxis (bleeding that arises from the nasal cavity) is a frequent occurrence in both ENT and accident/emergency departments globally and is one of the most prevalent emergencies [1]. It affects approximately 10-12% of the general population, with about 10% necessitating specialized medical intervention [2]. While epistaxis may arise from either anterior or posterior sources, nearly 90% of nasal bleeding cases can be attributed to Kiesselbach's plexus (also known as Little's area) located on the anterior portion of the nasal septum. Notably, many of these cases can be effectively managed in a home setting [3]. The nasal cavity features abundant vascularity, supplied by branches from both internal and external carotid arteries [4].

The etiology of epistaxis remains elusive in the majority of cases and can be categorized into two main groups: local causes associated with the nose, paranasal sinuses, and the nasopharynx, as well as systemic causes such as hypertension, blood dyscrasias, and the usage of anticoagulant medications [5]. Risk factors for epistaxis in children include nose picking, trauma, nasopharyngeal mass, bacterial nasal colonization, and allergic rhinitis, among others [6]. While certain cases of epistaxis may require medical intervention and hospitalization, most such instances are typically self-limiting and without profound implications. These milder cases can often be managed successfully with simple first aid measures, such as applying digital compression with the child's head tilted forward to avoid blood trickling back into the throat, packing with a piece of cotton, or cold compresses applied to the nose [7]. It is essential to have sufficient knowledge of appropriate first aid techniques to handle acute epistaxis without immediate access to hospital facilities, despite the high prevalence of this condition [2].

Recognizing the need for first aid management, parents must understand the steps involved in stopping the bleeding and providing comfort to their children. Recent studies have shown that while parents are generally aware of first aid measures for epistaxis, their knowledge and confidence in executing these measures remain suboptimal [8,9]. In a cross-sectional study conducted at government primary healthcare centers in Al-Madinah Al-Munawwarah, a province in Saudi Arabia, findings revealed that only 27.9% of parents accurately responded when asked about the correct course of action to take for a case of epistaxis [10]. Meanwhile, in other studies, parents were moderately knowledgeable about this issue [9,11]. Furthermore, many pervasive myths still exist surrounding epistaxis first-aid measurements [12]. These practices highlight the need for more accurate, evidence-based guidance. As no related studies were conducted in our region, Arar (Northern Borders area, Saudi Arabia), this study aims to explore the level of awareness among parents in our region concerning the first aid management of epistaxis. The results will underscore the importance of education in empowering parents to handle such situations.

## Materials and methods

Research design

This cross-sectional study was conducted in Arar, the capital of the Northern Borders region (with a population of about 373,577) and one of the 13 provinces in Saudi Arabia, which has been described in detail previously [13].

Inclusion criteria

The study included parents who (1) were residing in Arar and had at least one living child, (2) were aged 18 years or above, (3) could read and write in Arabic or English, and (4) were willing to participate in the study.

Exclusion criteria

The exclusion criteria included parents who were (1) residing outside Arar, Saudi Arabia, (2) aged less than 18 years, and (3) unwilling to participate in the study.

Sampling method and sample size calculation

A convenient sampling method was applied to recruit participants. The study was advertised through social media platforms, such as Facebook and Twitter. The minimum sample size for this study was calculated according to Swinscow as follows: n=z^2^p(1 - p)/d^2^, where n is the calculated sample size, z is the level of confidence according to the standard normal distribution (for a level of confidence of 95%, z = 1.96, p is the expected awareness of 27.9% based on Al-Johani et al. [10], 1 - p = 1 - 0.279 = 0.721, and d is the maximum acceptable error (= 0.05). So, the calculated minimum sample size was: n=(1.96)^2^ × 0.279 × 0.279/(0.05)^2^ = 309. The sample was increased to 340 by adding 10% to compensate for the incomplete questionnaires and dropouts.

Data collection and the related tool

All data were collected from mid-July to the end of September 2023 using an electronic self-administered questionnaire, which included a consent form, the sociodemographic and background items (15 questions), and the Epistaxis Knowledge-related items (12 questions). Each correct answer is given a score of one, and the maximum score is 12. The designed Arabic version of the questionnaire was prepared by reviewing the relevant literature [8-10], and a panel of experts in emergency medicine, first aid, and public health ensured its content validity to measure what it is intended to measure. The experts provided feedback on the relevance and clarity of the questions, and any raised concerns have been made accordingly. All collected data were anonymized during subsequent analysis.

Statistical analysis

The data obtained from the online survey were recorded in an Excel spreadsheet (Microsoft Corporation, Redmond, Washington, United States), assigned appropriate codes, and subsequently subjected to statistical analysis using IBM SPSS Statistics for Windows, Version 23 (Released 2015; IBM Corp., Armonk, New York). Descriptive statistics such as counts and percentages were used to summarize the information. Tables were employed to present the findings comprehensively. Moreover, the chi-square test was employed to examine associations between demographic variables and knowledge of first aid management of epistaxis, considering a p-value of less than 0.05 as an indicator of statistical significance.

Ethics considerations

The study was conducted following ethical principles and the Declaration of Helsinki regulations. Participants were informed about the purpose of the study, the risks and benefits of participating, and their right to withdraw at any time. Participants' confidentiality and anonymity were protected.

## Results

Basic characteristics of the study population

A total of 342 participants completed the questionnaire. Over half of the participants were 18-25 years old (n=184; 53.8%). Most participants were females (n=247; 72.2%), with the vast majority being married (n=30; 88.0%). Regarding the education level, 76.3% (n=261) of the participants had a college degree. Regarding occupation, more than half of the participants (n=175; 51.2%) were employed (Table 1).

**Table 1:** Sociodemographic characteristics of the study participants (n = 342) Data are presented as frequencies (n) and percentages (%).

Sociodemographic characteristics	Category	Frequency (%)
Age	18-25 years	184 (53.8%)
	26-35 years	65 (19.0%)
	36-45 years	59 (17.3%)
	46-55 years	15 (4.3%)
	56 years and above	19 (5.6%)
Sex	Male	95 (27.8%)
	Female	247 (72.2%)
Marital status	Married	301 (88.0%)
	Divorced	19 (5.6%)
	Widowed	22 (6.4%)
Education level	Less than high school	8 (2.3%)
	High school graduate	56 (16.4%)
	College graduate	261 (76.3%)
	Postgraduate degree	17 (5.0%)
Occupation	Employed	175 (51.2%)
	Self-employed	34 (9.9%)
	Unemployed	38 (11.1%)
	Student	87 (25.6%)
	Others	8 (2.3%)

The epistaxis background information of participants

As illustrated in Table 2, more than half of the respondents (n=223; 65.2%) had more than one child. Regarding the epistaxis experience of participants' children, the majority of the respondents' children (n=180; 52.6%) had never experienced epistaxis. Only around half of the participants' children (n=84; 51.9%) had received first aid management for epistaxis. A total of 114 respondents, accounting for 33.3% of the total sample, obtained knowledge on first aid management for epistaxis via online sources. Most participants felt it was important to know how to manage epistaxis through first aid. About 111 participants (32.6%) had ever attended any first aid training, with seventy-six (68.7%) feeling confident in managing epistaxis through first aid. More than half (n=192; 56.0%) of the respondents indicated they have never encountered difficulties managing epistaxis through first aid.

**Table 2:** Epistaxis background information of the participants Data are presented as frequencies (n) and percentages (%).

Epistaxis background information	Categories	Frequency (%)
Do you have more than one child (≥2 children)?	Yes (≥2 children)	223 (65.2%)
	No (just one child)	119 (34.8%)
Have your children ever experienced epistaxis?	Yes	162 (47.4%)
	No	180 (52.6%)
Have your child (children) ever received any first aid management for epistaxis?	Yes	84 (51.9%)
	No	78 (48.1%)
How did you learn about first aid management for epistaxis?	School	90 (26.4%)
	Healthcare professional	41 (12.1%)
	Family/friend	90 (26.2%)
	Internet	114 (33.3%)
	Others	7 (2.0%)
Do you think it is important to know how to manage epistaxis through first aid?	Yes	328 (95.8%)
	No	14 (4.2%)
Have you ever attended any first-aid training?	Yes	111 (32.6%)
	No	231 (67.4%)
How confident are you in managing epistaxis through first aid?	Very confident	76 (68.7%)
	Somewhat confident	28 (25.4%)
	Not confident	7 (5.9%)
Have you ever discussed epistaxis and its first aid with your family members?	Yes	229 (66.9%)
	No	113 (33.1%)
How likely are you to seek medical attention for epistaxis?	Very likely	127 (37.4%)
	Somewhat likely	150 (43.9%)
	Not likely	64 (18.7%)
Have you ever encountered any difficulties in managing epistaxis through first aid?	Yes	150 (44.0%)
	No	192 (56.0%)

Participants' awareness of first aid management of epistaxis

Table 3 presents the study findings on participants' awareness of the first aid management of epistaxis. Regarding the potential causes of epistaxis, the respondents provided mixed responses. When asked about the most common cause, 39.5% correctly identified trauma to the nose, while the majority of the respondents (41.9%) mentioned high blood pressure, 11.9% mentioned allergies, and only 6.7% cited dry air.

**Table 3:** Study participants' awareness of first aid management of epistaxis Data are presented in frequencies (n) and percentages (%).

Questions	Answers	Frequency (%)
What is the most common cause of epistaxis?	High blood pressure	143 (41.9%)
	Allergies	41 (11.9%)
	Dry air	23 (6.7%)
	Trauma to the nose	135 (39.5%)
Which of the following should you NOT do if your child has an epistaxis?	Pinch the nostrils together	57 (16.6%)
	Leaning the head back	138 (40.4%)
	Applying an ice pack to the nose	42 (12.4%)
	Sitting upright and leaning forward	105 (30.7%)
How long should the application pressure to the nose if your child has an epistaxis?	5 minutes	132 (38.7%)
	10 minutes	78 (22.8%)
	15 minutes	110 (32.3%)
	20 minutes	21 (6.2%)
Which of the following can help prevent epistaxis?	Drinking plenty of water	16 (4.8%)
	Using a humidifier	98 (28.6%)
	Avoiding picking the nose	94 (27.4%)
	All of the above	134 (39.2%)
What you should do if your child has frequent or severe epistaxis?	See a doctor	326 (95.2%)
	Apply ice to the nose	8 (2.4%)
	Using a nasal spray	4 (1.2%)
	None of the above	4 (1.2%)
What is the first step in managing epistaxis?	Pinching the nostrils together	57 (16.6%)
	Leaning the head back	101 (29.4%)
	Sitting upright and leaning forward	177 (51.7%)
	Applying an ice pack to the nose	8 (2.3%)
What is the most common cause of recurrent epistaxis?	High blood pressure	143 (41.9%)
	Allergies	41 (11.9%)
	Dry air	23 (6.7%)
	Trauma to the nose	135 (39.5%)
Which of the following should you NOT do if your child has recurrent epistaxis?	Using a nasal spray	57 (16.6%)
	Taking aspirin or other non-steroidal anti-inflammatory drugs without medical advice	138 (40.4%)
	Blowing the nose forcefully	42 (12.4%)
	Drinking plenty of fluids	105 (30.7%)
What is the most severe complication of epistaxis?	Anemia	143 (41.7%)
	Infection	46 (13.4%)
	Loss of consciousness	102 (29.8%)
	None of the above	52 (15.1%)
How can you prevent epistaxis if your child is prone to it?	Using a humidifier	53 (15.6%)
	Applying a saline nasal spray	69 (20.3%)
	Avoiding picking the nose	83 (24.4%)
	All of the above	136 (39.7%)
What should you do if the epistaxis does not stop after 20 minutes of applying pressure?	See a doctor	323 (94.3%)
	Applying ice to the nose	5 (1.6%)
	Using a nasal spray	10 (2.9%)
	None of the above	4 (1.2%)
How often should you change the gauze or tissue you are using to absorb blood during epistaxis?	Every 5 minutes	138 (40.5% )
	Every 10 minutes	107 (31.4%)
	Every 15 minutes	89 (26.1%)
	Every 20 minutes	7 (2.0%)

Only 40.4% of the respondents correctly selected the step that should not be taken for first aid management of epistaxis: "leaning the head back." Regarding the duration of intervention for applying pressure on the nose, only 22.85% of respondents correctly stated the time of 10 minutes. Regarding what can help prevent epistaxis, 39.2% of the respondents correctly stated all the measures, including drinking plenty of water, using a humidifier, and avoiding picking the nose. About 95.2% of the respondents noted that one should see a doctor if their child (children) experienced frequent or severe epistaxis. Regarding the first step in managing epistaxis, more than half of the respondents (51.7%) selected the correct state: sitting upright and leaning forward.

Regarding recurrent epistaxis, when asked about its common cause, 39.5% correctly identified trauma to the nose. Additionally, 41.7% of the respondents correctly identified anemia as the most severe complication of epistaxis. One hundred thirty-six of the respondents (39.7%) correctly stated using a humidifier, applying a saline nasal spray, and avoiding picking the nose as measures of preventing recurrent epistaxis. Regarding the timelines for action, 94.3% of the respondents correctly stated that if bleeding does not stop within 20 minutes of applying pressure, the patient must see a doctor.

Association of participants' demographic data and knowledge of epistaxis first aid management

Table 4 depicts the relationship between age, sex, level of education, and occupation, and the knowledge of first aid management of epistaxis. The results established a significant association between the knowledge of first aid management of epistaxis and age (p = 0.002), sex (p = 0.003), and education level (p = 0.001). Meanwhile, no significant association was evident with occupation (p > 0.05).

**Table 4:** The association between age, gender, level of education, occupation, and knowledge of first aid management of epistaxis The chi-square test was applied. Significance was set at p < 0.05.

Variables	Category	Knowledge of first aid management of epistaxis		
		High	Low	p-value
Age	18-25 years	56.4%	43.6%	0.002
	26-35 years	57.9%	42.1%	
	36-45 years	57.5%	42.5%	
	46-55 years	57.3%	42.7%	
	56 years and above	58.2%	41.8%	
Sex	Male	43.1%	56.9%	0.003
	Female	56.4%	43.6%	
Education level	Less than high school	56.6%	43.4%	0.001
	High school graduate	57.3%	42.7%	
	College graduate	59.4%	40.6%	
	Postgraduate degree	56.5%	43.5%	
Occupation	Employed	41.4%	58.6%	0.139
	Self-employed	43.6%	56.4%	
	Unemployed	43.5%	56.5%	
	Student	42.1%	57.9%	
	Others	44.%	56.%	

## Discussion

Nosebleeds, also known as epistaxis, are a common medical concern worldwide, and Saudi Arabia is no exception. In Saudi Arabia, epistaxis has been reported as a frequent complaint in emergency rooms and primary healthcare settings. However, despite its prevalence, epistaxis often receives inadequate attention, as it is often considered a minor ailment [14]. This notion is problematic, as epistaxis can result in significant morbidity and may occasionally signify underlying medical conditions. Therefore, understanding the general awareness of epistaxis within our region and globally is imperative to improve public knowledge, prioritize comprehensive management strategies, and mitigate potential complications associated with this seemingly benign condition.

Our study aims to assess the awareness level concerning the first aid management of epistaxis among parents in Arar, Saudi Arabia. The participants for this study predominantly comprise young adults between the ages of 18 and 25, with a higher representation of females. Furthermore, the participants exhibit relatively advanced educational backgrounds and are primarily employed. According to the findings, only 47.4% of the participants' children reported prior experience with epistaxis, substantially lower than the 60% global prevalence rate of epistaxis in the general population. This suggests a possible underreporting due to self-treatment and the perception of nosebleeds as a trivial event not requiring medical care [15].

The findings revealed that 32.6% of the participants had received first aid training for epistaxis management, while about one-third (33.3%) learned through the Internet. These findings are similar to those from other studies conducted in Saudi Arabia (Table 5). The study by Alshehri et al. found that 67% of the study participants had experienced epistaxis, and 54% had received information about first aid for epistaxis in Alahssa, Saudi Arabia [16]. In another study, Almutairi et al. found that 79.9% of the people had experienced epistaxis at least once in their lifetime in Al-Majmaah, Saudi Arabia [17]. Based on our findings, there is a significant knowledge gap on common medical emergencies that should be addressed through public first aid education and public health initiatives.

**Table 5:** Characteristics of studies in Saudi Arabia concerning assessment of awareness level of first aid management of epistaxis PHCs: primary health centers, KAUH: King Abdul-Aziz University Hospital.

Study	Study location	Sample size	Data collection protocol	Reported outcome(s)
Almuulhim, 2017 [11]	Saudi Arabia	1114	Distributing questionnaires to the general population	- Approximately 67.4% of the participants were knowledgeable about the management of epistaxis
Al-Johani, 2018 [10]	Al-Madinah Al-Munawwarah	390	Self-administered questionnaires to the parents attending PHCs	- The study revealed that 27% of parents indicated that the initial treatment for epistaxis involves applying pressure while tilting the head downwards.
Alshehri, 2018 [16]	Alahssa	485	Distributing questionnaires electronically to the school teachers	- Out of the teachers surveyed, 54% of the participants had received information regarding first aid to stop epistaxis. Additionally, 67% of teachers reported that their students had previously experienced epistaxis. About 15% of teachers stated that they would not attempt to stop the bleeding, while only 25% mentioned they would apply pressure on the cartilaginous part of the nose. However, a higher percentage (57%) correctly understood that tilting the head forward is the appropriate action to take.
Al-Kubaisy, 2019 [18]	Riyadh	1,073	Questionnaire distribution concerning the international guidelines for first aid management of epistaxis to school teachers	- About 85.5% of the teachers in the sample were female. - As for the awareness and knowledge about epistaxis and related interventions, one-third of the teachers were well-informed. - The highest level of awareness was related to head posture and nose pressure.
Alyahya, 2019 [2]	Riyadh	300	Questionnaire distribution electronically to medical students in college	- A significant majority of the participants (75.7%) were female, indicating a gender imbalance among the respondents. - Medical students possess a satisfactory understanding of the first aid and management techniques for epistaxis. - Self-education emerged as the primary source of knowledge acquisition for the respondents regarding epistaxis first aid and management.
Abu Suliman, 2020 [14]	Saudi Arabia	1475	Distributing questionnaires electronically to the general population	- Out of the participants, 81% had a previous experience of epistaxis. - The collective level of knowledge was found to be 64%. - A significant 52.2% of the participants held the belief that adjusting the position of their head could halt the bleeding.
Alasiri, 2022 [19]	Asser	382	Distributing questionnaires electronically to teachers in schools	- Concerning epistaxis and how to handle it, approximately two-thirds of the teachers were informed. Those who taught scientific subjects, those who had received education on the topic, and young male teachers exhibited higher levels of understanding.
Merdad, 2022 [20]	Jeddah	131	Distributing questionnaires electronically randomly through WhatsApp to all healthcare providers and medical students at KAUH	- The healthcare providers possess limited knowledge regarding appropriate first aid measures for epistaxis. - Approximately 30% of participants correctly identified the necessary steps for providing initial care in such situations. - Moreover, only 30% of respondents accurately recognized the proper location for nasal compression, while 66% correctly positioned the patient's head. - Notably, the Oto-HNS specialists received the highest rating regarding their understanding of epistaxis first aid measures.
Alzahrani, 2023 [21]	Al-Baha	439	Distributing questionnaires electronically to teachers in schools	- No correlation was found between age, sex, or employment status and training effectiveness for managing epistaxis. However, teachers with a scientific background demonstrate a higher proficiency in providing effective management solutions
Alshehri, 2023 [22]	Saudi Arabia	2441	Distributing questionnaires electronically to the general population	- Out of the total respondents, 80% know that epistaxis is a chronic condition. - Most of this awareness comes from a large age group from 19 to 29.
Alotaibi, 2023 [23]	Hail	824	Distributing questionnaires electronically to medical students in college	- Students from Hail University showed a moderate level of comprehension regarding treating epistaxis. There is comparable knowledge between medical students and those pursuing other fields, and out of all the participants, 71% reported personally experiencing epistaxis.
Bashekah, 2023 [24]	Saudi Arabia	1135	Distributing questionnaires electronically to the general population	- Out of 8 (55.0%), the study participants' mean knowledge score was 4.4 (SD: 2.8). - First aid information was most frequently reported from the media (37.6%). - Knowledge and attitudes about first aid were impacted by gender, education, and socioeconomic level.
Alam, 2023 [9]	Taif	502	Distributing questionnaires to parents who visited Alhada Armed Forces Hospital and Prince Mansour Military Hospital	- Around 30.9% of parents exhibited a high level of understanding, while the remaining 67.5% displayed a moderate level of knowledge. - Several factors were linked to the parents' level of expertise, including the number of children they had, their age, whether they attended first aid classes, and their previous experience treating individuals with epistaxis.
Alanazy, 2023 [25]	Qassim	1152	Distributing questionnaires electronically as a Google form to school teachers	- Out of all the participants, 80% demonstrated a low level of knowledge. - It was found that being female and receiving accurate information about first aid for epistaxis were both factors associated with a higher knowledge score.
Mahzara, 2023 [8]	Jazan	622	Distributing questionnaires electronically to the general population	- The research findings indicated that a mere 40% of the participants possessed knowledge about the necessary measures to manage epistaxis using first aid techniques. Additionally, the study also highlighted a deficiency in knowledge concerning the causes and risk factors associated with nosebleeds.
Almutairi, 2023 [17]	Al Majmaah	407	Distributing questionnaires electronically to the general population	- A total of 325 reported experiencing epistaxis at least once in their lifetime. The results indicated that 94% of the population knew the correct position for controlling bleeding, with sitting tilted forward (48.9%) and backward (28.7%) being the commonly practiced coping mechanism. The study concluded that the general population possessed sufficient knowledge of epistaxis management, reflecting positively on the healthcare system, and recommended further research with a larger sample size to validate their findings.
Shosho, 2023 [26]	Makkah	1259	Distributing questionnaires electronically to the general population	- A majority of participants showed insufficient understanding of proper first aid for epistaxis, as indicated by only 37.1% demonstrating adequate knowledge. Regarding demographics, women, healthcare professionals, and individuals who had experienced nosebleeds in the past exhibited a higher likelihood of having good knowledge than others. - The primary sources of knowledge cited were relatives, friends, and social media rather than healthcare providers.

Currently, the overall knowledge about first aid for epistaxis was modest, with slightly more than half of the participants demonstrating adequate awareness. However, it is worth noting that with convenient sampling and possibly more educated parents responding, there is a high chance this number in the general population will be low. Most of the participants (39.5%) identified trauma to the nose as the common cause of epistaxis. Regarding first aid, less than half knew what they should not do if their children have experienced epistaxis: leaning the head back. The study noted substantial knowledge about the duration of intervention, preventive measures, and appropriate initial management, as well as the emergency responses for epistaxis and its recurrence. In agreement with our findings, other studies concluded similar results (Table 5). Also, the present findings agree with the meta-analysis done by Alkhalifah et al., in which they found that "the average awareness level of epistaxis and its management was approximately 63%, indicating a moderate level of awareness among Saudi residents". The researchers concluded that "gender, prior knowledge, and profession were significant factors that could influence the levels of awareness observed in their analysis" [27].

Study limitations

The significant limitation of this study was the employment of observational cross-sectional design, limiting the ability to establish cause-and-effect relationships and challenging to assess changes or trends. It also may not account for confounding variables or control for potential biases. Secondly, since the study utilized electronic questionnaires to collect data, it was challenging to discover insights or observe the behavior of respondents in their natural settings and the potential for recall bias, as participants may not accurately recall past events or behaviors. Also, the study findings cannot be generalized to the entire Saudi Arabian population, considering that it was conducted in only one region.

## Conclusions

The present study discovered that parents living in Arar, Saudi Arabia, have a limited understanding of how to handle epistaxis when it comes to first aid. The level of awareness was influenced by various factors associated with the demographic characteristics of the participants in the study. These findings strongly emphasize the necessity to address the identified knowledge gap through targeted educational initiatives, workshops, or public health campaigns. Empowering parents with the necessary knowledge and skills to provide prompt and appropriate first aid to their children and others who may experience epistaxis is warranted. Significantly, improving first aid readiness not only enhances overall health outcomes but also contributes to building a resilient and prepared community. Through this work, we can ultimately reduce the potential risks associated with epistaxis and ensure the well-being of individuals in our region.
